# Comparative genomics of quinolone-resistant *Escherichia coli* from broilers and humans in Norway

**DOI:** 10.1186/s12866-024-03412-3

**Published:** 2024-07-06

**Authors:** Jannice Schau Slettemeås, Camilla Sekse, Marianne Sunde, Madelaine Norström, Astrid Louise Wester, Umaer Naseer, Gunnar Skov Simonsen, Charlotte Rosenberg Ulstad, Anne Margrete Urdahl, Karin Lagesen

**Affiliations:** 1 Norwegian Veterinary Institute, P.O. box 64, Ås, 1431 Norway; 2 Norwegian Institute of Public Health, P.O. box 4404, Nydalen, Oslo, 0403 Norway; 3 University Hospital of North Norway, Breivika, Tromsø, 9038 Norway; 4 Faculty of Health Sciences, UiT – The Arctic University of Norway, Tromsø, Norway; 5 Department of Microbiology and Infection Control, Akershus University Hospital, Lørenskog, Norway

**Keywords:** Antimicrobial resistance, Quinolone resistance, *Escherichia coli*, Human, Broiler, Core genome phylogeny

## Abstract

**Background:**

The usage of fluoroquinolones in Norwegian livestock production is very low, including in broiler production. Historically, quinolone-resistant *Escherichia coli* (QREC) isolated from Norwegian production animals rarely occur. However, with the introduction of a selective screening method for QREC in the Norwegian monitoring programme for antimicrobial resistance in the veterinary sector in 2014; 89.5% of broiler caecal samples and 70.7% of broiler meat samples were positive. This triggered the concern if there could be possible links between broiler and human reservoirs of QREC. We are addressing this by characterizing genomes of QREC from humans (healthy carriers and patients) and broiler isolates (meat and caecum).

**Results:**

The most frequent mechanism for quinolone resistance in both broiler and human *E. coli* isolates were mutations in the chromosomally located *gyrA* and *parC* genes, although plasmid mediated quinolone resistance (PMQR) was also identified. There was some relatedness of the isolates within human and broiler groups, but little between these two groups. Further, some overlap was seen for isolates with the same sequence type isolated from broiler and humans, but overall, the SNP distance was high.

**Conclusion:**

Based on data from this study, QREC from broiler makes a limited contribution to the incidence of QREC in humans in Norway.

**Supplementary Information:**

The online version contains supplementary material available at 10.1186/s12866-024-03412-3.

## Introduction

Fluoroquinolones are a group of broad-spectrum antimicrobials that have been used for treatment of a wide range of infections in both humans and animals. In humans there are multiple approved indications for the use of fluoroquinolones, among them bone and joint infections, gastrointestinal infections, urinary tract infections (UTI) and respiratory tract infections [1]. However, the human use of this group of antimicrobials is very low in Norway compared to other countries, and primarily restricted to patients with complicated urinary tract or intra-abdominal infections [2–4]. Fluoroquinolones have since 2019 been classified by the World Health Organization as critically important for human treatment [5], and have been restricted for use in animals by the EU Antimicrobial Advise ad hoc Expert Group (AMEG) since 2020 [6]. From 1993 to 2021 the proportion of sales of fluoroquinolones for food-producing terrestrial animals in Norway varied between 0.1% and 0.3%, while in humans the quinolone usage has decreased by more than 60% since 2012 [7]. This restrictive use of fluoroquinolones is reflected through low occurrence of quinolone-resistant *Escherichia coli* (QREC) in the national surveillance data on antimicrobial resistance, i.e. in the monitoring programme for antimicrobial resistance in bacteria from feed, food and animals (NORM-VET) and in the surveillance programme for antimicrobial resistance in human pathogens (NORM).

Quinolone resistance among *E. coli* from broilers investigated in NORM-VET has historically been very low, though with an increase from 3.4% in 2014 to 12.6% in 2020 [8–11]. These data are based on a non-selective method, where one indicator/random *E. coli* isolate is selected from one pooled caecal sample from ten broilers per flock, and then further susceptibility tested and interpreted using epidemiological cut-off values (ECOFFs) as defined by the European Committee on Antimicrobial Susceptibility Testing (EUCAST) [12]. In *E. coli* isolates from human blood and UTIs in Norway in 2014, the occurrence of fluoroquinolone resistance based on clinical breakpoints was 12.6% and 8.7% of all isolates tested, respectively [8]. Since then, quinolone resistance seems to have been relatively stable between 10% and 13% in *E. coli* from blood and between 7.5% and 9% in *E. coli* from urine samples [13].

Fluoroquinolones act by inhibiting DNA synthesis of two essential type II topoisomerases, DNA gyrase and topoisomerase IV [14–16]. The mechanisms of quinolone resistance are either chromosomal point mutations in genes causing a reduced affinity to protein targets, reduced accumulation of drugs either by a decrease in uptake or an increase in efflux of the agent, or plasmid-mediated quinolone resistance (PMQR) genes encoding target protection [14]. Chromosomal point mutations are located within a small quinolone resistance-determining region (QRDR) of the genes *gyrA*, *gyrB*, *parC* and *parE*. The level of resistance increases with the number of mutations in these genes [14]. For *E. coli*, the most amino acid substitutions are at the Ser83 (S83) and Asp87 (D87) in GyrA and at Ser80 (S80) and Glu84 (E84) in ParC. The product of plasmid-located *qnr* genes, on the other hand, is a pentapeptide repeat family molecule that blocks the action of ciprofloxacin on DNA gyrase and topoisomerase IV [17, 18]. Several plasmid-located gene variants have been described; *qnrA*,* qnrB*, *qnrC*, *qnrD*, *qnrE*, *qnrS*, *qnrVC*, as well as *aac(6’)-lb-cr*, *oqxAB* and *qepA* [16, 19–21].

Some *E. coli* might cause extraintestinal disease such as UTIs, sepsis and respiratory tract infections and have been identified as extraintestinal pathogenic *E. coli* (ExPEC) [22]. A common method for characterizing *E. coli* is multi-locus sequence typing (MLST), molecular typing based on seven housekeeping genes, which is used for identifying and tracking specific sequence types (STs) that are associated with antimicrobial resistance and/or pathogenicity [22, 23].

Some *E. coli* STs have frequently been identified as human ExPEC including ST10, ST69, ST73, ST95, ST117, and ST131 [24, 25]. Moreover, some successful ExPEC STs, disseminated worldwide, have been detected as antimicrobial resistant lineages both from humans and from food/animal sources, like ST10, ST69, ST95, ST117, ST131, and ST405 [24, 26–29]. In a review from Manges [24], poultry and poultry meat was hypothesized as a reservoir for human ExPEC. This was further supported by a Swedish study [30, 31], which showed that identical or closely related AmpC or extended-spectrum beta-lactamase (ESBL)-producing *E. coli* assigned to ST10, ST38, ST69 and ST131 were found both on Swedish and imported chicken meat. The Swedish study indicates both that the introduction of the AmpC-/ESBL resistant *E. coli* strains in broiler was due to broiler import and that faecal contamination to meat occurring at slaughter represents a source for further spread to humans. Also, a recent Italian study described various human pandemic and emerging ExPEC STs such as ST10, ST23, ST69, ST95, ST117 and ST131 from poultry [28]. In that study, the quinolone resistance mechanisms detected in approximately 45–50% of the quinolone-resistant isolates were mainly due to chromosomal point mutations in *gyrA*, *parC* and/or *parE*, though *qnrS1* and *qnrB19* were also detected. Moreover, a study on fluoroquinolone-resistant avian pathogenic *E. coli* (APEC) from Korea identified chromosomal point mutations in *gyrA*, *parC* and *parE* as the main reason behind the quinolone resistance mechanism, while *qnrS1* was the only PMQR gene detected in only a few isolates [29]. Some of these isolates were assigned to major lineages of ExPEC such as ST95 (*n* = 3) and ST69 (*n* = 2). One or more mutations in *gyrA*, *parC* or *parE* were identified in these isolates, in addition, two of the ST95 isolates carried the *qnrS1* gene.

The aim of the present study was to explore whether QREC from broilers in Norway may contribute to the occurrence of QREC in humans. We collected, whole genome sequenced and compared isolates that were phenotypically resistant to quinolones. They were from both humans and broilers, 100 isolates from each. Broiler QREC isolates were selected in a manner that ensured representativeness among QREC in broilers in Norway. The human QREC isolates were from UTI or bacteraemia isolates, as well as isolated from stool samples from healthy carriers. We performed whole genome sequencing followed by MLST analysis, detection of genes associated with quinolone resistance and other antimicrobial resistance genes as well as phylogenetic analysis.

## Results

### Isolate overview

In total, 200 QREC genomes were sequenced and assembled. Ten of the human isolates were excluded, seven because they were phenotypically susceptible to ciprofloxacin and/or nalidixic acid, two due to low quality of sequences/reads, and one that turned out to be *Klebsiella variicola* (Mash Screen). The reads as well as the annotated assemblies were deposited in the European Nucleotide Archive (ENA) repository under study accession numbers PRJEB36302 and PRJEB33048 and at the Sequence Read Archive (SRA) under BioProject accession number PRJNA1117742 (Additional file 1). On average, the assemblies contained 111 contigs (min: 39, max: 491), and had an average N50 size of 173,710 bp (min: 57,055, max: 361,993). Details regarding the quality of the genome data are available in the MultiQC and Quast reports provided in Additional file 2 and 3, respectively.

All 190 isolates included in the analysis were either resistant to ciprofloxacin (*R* > 0.06 mg/L) and/or nalidixic acid (*R* > 8 mg/L) according to ECOFFs and inclusion criteria set for the study. The overall MIC distributions and the antimicrobial resistance profiles for both the broiler and human isolates are shown in Additional file 1. In total, 68 of the 90 human isolates (75.6%) were considered multidrug resistant (MDR) (i.e. resistant to three or more antimicrobial classes), of which 42 were isolated from bacteraemia and twelve from UTI cases. Among the 100 broiler isolates 23 (23%) were considered MDR, of which eleven were isolated from meat and twelve from caecum.

### Quinolone resistance genes

#### Point mutations

Table 1 gives an overview of the detected chromosomal point mutations causing quinolone resistance in all the 190 QREC isolates. A detailed description of the mutations in each isolate is shown in Additional file 1.

**Table 1:** Overview of the chromosomal point mutations causing quinolone resistance by amino acid substitution in the *Escherichia coli* isolates (*n* = 190) from broilers and from humans

	# of isolates	# of mutations	GyrA	GyrB	ParC	ParE
Broiler	65	1	S83L			
	3	1	D87N			
	1	1				T492C
	22	2	S83L			D475E
	8	2	S83L, D87N			
	1	3	S83L			L488M, A512T
Human	9	0				
	42	1	S83L			
	2	1	D87G			
	1	1	S83A			
	5	2	S83L, D87N			
	2	2	S83L			S458A
	2	2	S83L			A426V
	2	2	S83L			D475E
	1	2	S83L		S58I	
	1	2	S83L		E84G	
	9	3	S83L, D87N		S80I	
	1	3	S83L, D87G		S58I	
	1	3	S83L, D87N		S80R	
	1	3	S83L, D87N		S58I	
	1	3	S83W	V467L	E84K	
	4	4	S83L, D87N		S58I	L416F
	3	4	S83L, D87N		S58I, S80I	
	1	4	S83L, D87N		S80I	S458A
	1	5	S83L, D87N		S58I, S80I	L416F
	1	5	S83L, D87N		S58I, S80I	E460K

Overall, amino acid substitutions were more diverse in the human than in the broiler QREC isolates (16 different substitutions in all four genes and six different substitutions altogether in two genes, respectively). In broiler QREC isolates compared to human isolates, point mutations in one gene were more common (*gyrA* (*n* = 76) or *parE* (*n* = 1)) followed by two genes (*gyrA* + *parE* (*n* = 23)). The human isolates were more diverse when it came to point mutations: no mutations (*n* = 9), point mutations in *gyrA* only (*n* = 52), point mutations in two genes (*gyrA* + *parC* (*n* = 17) or *gyrA* + *parE* (*n* = 4)), or point mutations in three genes (*n* = 8). Interestingly, none of the broiler isolates had substitutions in *gyrB* nor *parC*. For both broiler and human isolates, the amino acid substitution S83L in GyrA were most frequent (*n* = 96 and *n* = 77, respectively). Thereafter, the most frequent amino acid substitutions D475E in ParE (*n* = 22) and D87N in GyrA (*n* = 11) among the broiler isolates, and D87N in GyrA (*n* = 26), S80I in ParC (*n* = 15) and S58I in ParC (*n* = 15) among the human isolates.

### Acquired genes

In total, five plasmid-located quinolone resistance genes were detected; five in broiler and 13 in human QREC isolates (Table 2). Two of the genes (*qnrB19* and *qnrS1*) were found in both human and poultry isolates, while the remaining three genes (*aac(6’)-Ib-cr*, *qnrB1* and *qnrB5*) were found in human isolates, only. An overview of the total acquired genes detected in the QREC isolates are shown in Additional file 1.

Of the five broiler isolates with acquired quinolone resistance genes, four carried the *qnrS1* gene together with the amino acid substitution S83L in GyrA, while the last one carried the *qnrB19* gene together with the same amino acid substitution S83L in GyrA. The *qnrS1* gene was also detected in five (35.7%) of the 13 human isolates, but only one had the amino acid substitution S83L in GyrA, and none had the same sequence type (ST) as the *qnrS1* carrying broiler isolates. Four (28.6%) of the human isolates carried the *qnrB19* gene, of which one had three amino acid substitutions at S83L and D87N in GyrA and S80I in ParC, and none had the same ST as that of the *qnrB19* carrying broiler isolates.

For acquired genes that were exclusively found in human isolates, the *aac(6’)-lb-cr* gene was detected in three (21.4%) isolates. One isolate had the additional amino acid substitution S83L in GyrA and another one had two amino acid substitutions (S83L in GyrA and S58I in ParC), and one isolate contained *qnrB1* but no additional amino acid substitutions. The last isolate contained the *qnrB5* gene together with three amino acid substitutions at S83L and D87N in GyrA and S80I in ParC.

**Table 2:** Quinolone resistance mechanisms and sequence types among broiler (*n* = 5) and human (*n* = 13) isolates carrying acquired quinolone resistance genes

Specimen		Sample ID	CIP* MIC mg/L	NAL* MIC mg/L	GyrA	ParC	No. of mutations	Acquired gene	Sequence type (ST)
BROILER	meat	2014-01-5749	2	256	S83L		1	*qnrS1*	ST453
	caecum	2014-01-6924	1	64	S83L		1	*qnrS1*	ST453
	caecum	2014-01-7234	1	64	S83L		1	*qnrS1*	ST453
	caecum	2014-01-7375	2	128	S83L		1	*qnrS1*	ST453
	caecum	2014-01-5792	0.25	64	S83L		1	*qnrB19*	ST349
HUMAN	blood	Q03-42	4	256	S83L	S58I	2	*aac(6’)-Ib-cr*	ST131
	blood	Q03-33	1	128	S83L		1	*aac(6’)-Ib-cr*	ST136
	blood	Q02-32	4	64			0	*aac(6’)-Ib-cr*,* qnrB1*	ST12
	blood	Q02-58	0.50	8			0	*qnrB19*	ST778
	stool	E8-09	0.25	8			0	*qnrB19*	ST10
	stool	E7-51	0.12	4			0	*qnrB19*	ST3877
	UTI*	Q01-81	16	256	S83L, D87N	S80I	3	*qnrB19*	ST162
	UTI*	Q04-11	8	256	S83L, D87N	S80I	3	*qnrB5*	ST162
	blood	Q02-39	1	16			0	*qnrS1*	ST10
	blood	Q02-30	0.25	4			0	*qnrS1*	ST14
	blood	Q01-38	0.50	8			0	*qnrS1*	ST4434
	stool	E4-64	0.25	32			0	*qnrS1*	ST10
	stool	E7-61	4	256	S83L		1	*qnrS1*	ST117

*CIP – ciprofloxacin; NAL – nalidixic acid; UTI – urinary tract infection

### Multi-locus sequence types (MLST) and phylogenetic analysis

**Fig. 1:**
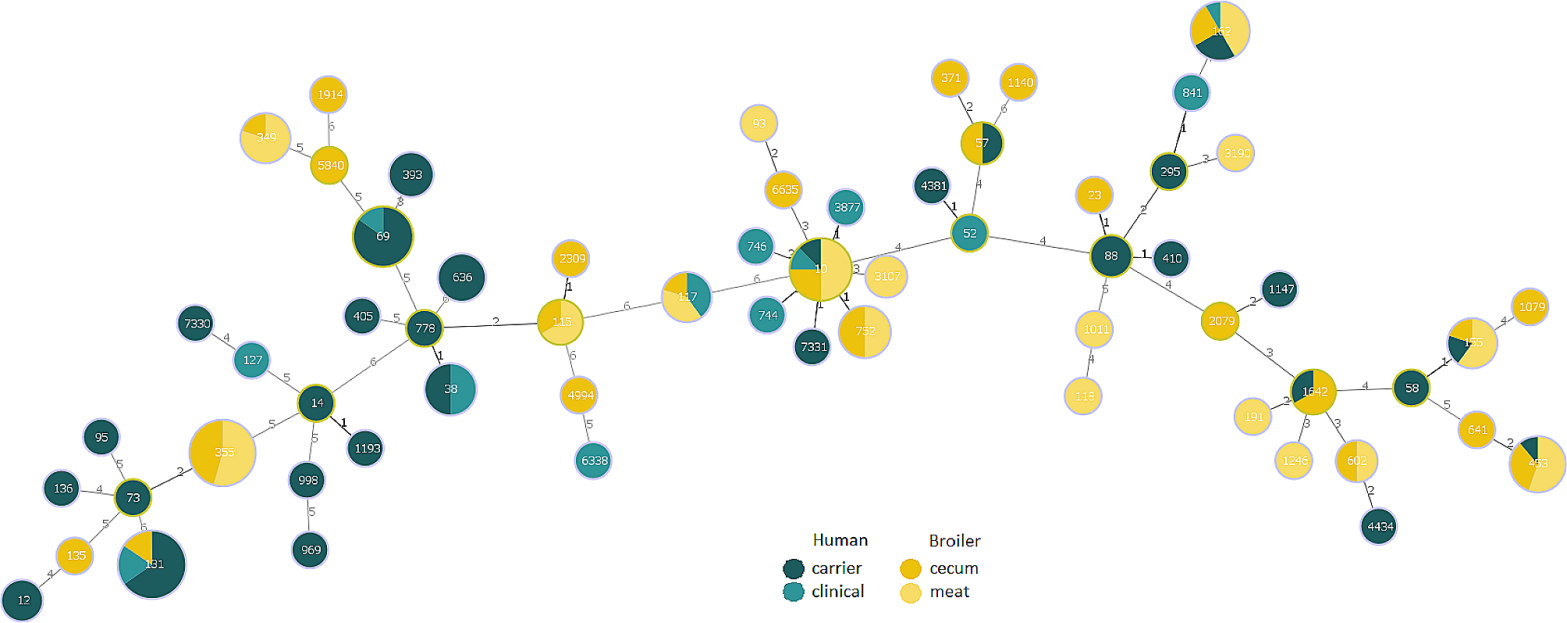
eBURST analysis. Minimum Spanning Tree of all MLSTs using PHYLOViZ v 2.0 showing the clonal relationship of broiler (*n* = 100) and human (*n* = 90) quinolone-resistant *Escherichia coli* isolates. Size of the circle corresponds to the number of isolates. The colour of the circle borders corresponds to group founder (light green) and common node (light blue)

In total, the QREC isolates were assigned into 62 unique *E. coli* MLSTs, as illustrated in Fig. 1. The human QREC isolates were assigned into 38 STs, where 62 isolates grouped into ten different STs and 28 were singletons. Broiler QREC isolates where assigned to 32 STs, 81 isolates grouped into 13 different STs and 19 were singletons. The most prevalent *E. coli* STs were ST131 (*n* = 26, 22 human, 4 broiler), ST355 (*n* = 22, all broiler), ST10 (*n* = 16, 4 human, 12 broiler), ST69 (*n* = 13, all human), ST162 (*n* = 12, 4 human, 8 poultry), and ST453 (*n* = 9, 1 human, 8 poultry). In total, 30 different STs were exclusively found in human isolates, 24 were found in broiler isolates, only, while eight STs were identified in both species (STs 453, 155, 1642, 162, 57, 10, 117 and 131).

**Fig. 2:**
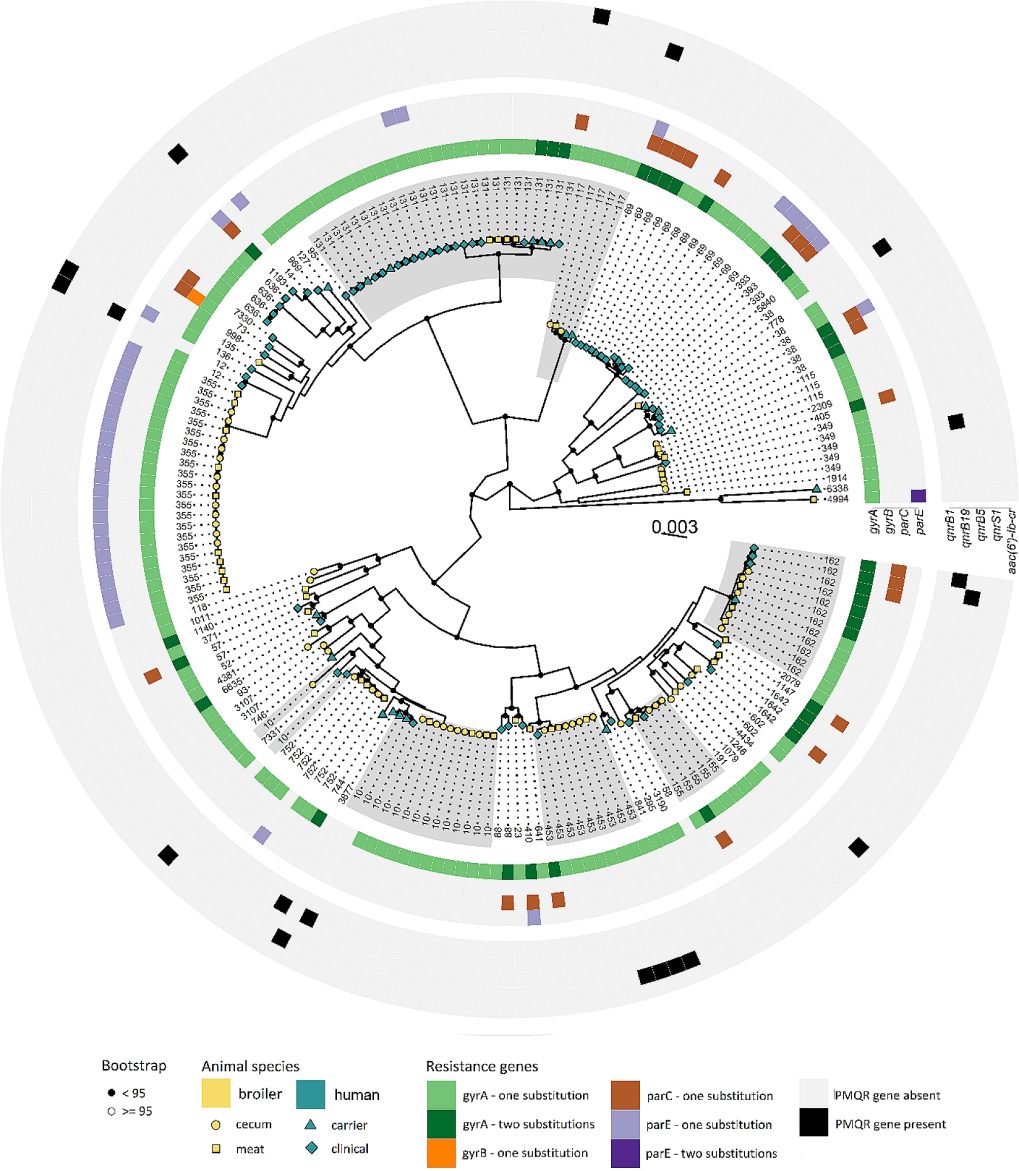
Maximum likelihood SNP tree of 100 broiler and 90 human quinolone-resistant *Escherichia coli* isolates. Branch supports (ultrafast bootstrap approximation) are denoted with black or white nodes. The coloured tips and shapes on the tree denote species of origin and material. The tip labels denote the sequence types from the MLST scheme hosted by EnteroBase. The colouring on the outer rings denotes the presence/absence of mutations leading to amino acid (AA) substitutions in chromosomal genes and the presence/absence of plasmid-mediated genes leading to quinolone resistance. The grey shades denote groups of STs that contains isolates from both hosts, and which contains five or more isolates in total, ST10, ST117, ST131, ST155, ST162, and ST453. The tree was generated with IQTree from SNPs in core genes from Roary aligned with MAFFT. The evolutionary model used was GTRFASCR9 - GTRGAMMA. The tree is midpoint rooted for better visualization

A maximum likelihood tree based on core gene SNPs was made (Fig. 2) to further illustrate the relationship between isolates. The pangenome analysis of the 190 genomes detected 19 853 unique genes. Of these, 3156 were defined as core genes, i.e. present in at least 95% of the genomes. As expected, isolates with the same ST predominantly clustered closely together, with the notable exception of ST10. The STs that contained isolates from both species did not display any within-cluster clade separation between isolates stemming from the two species.

### Core genome phylogeny on selected sequence types

**Fig. 3:**
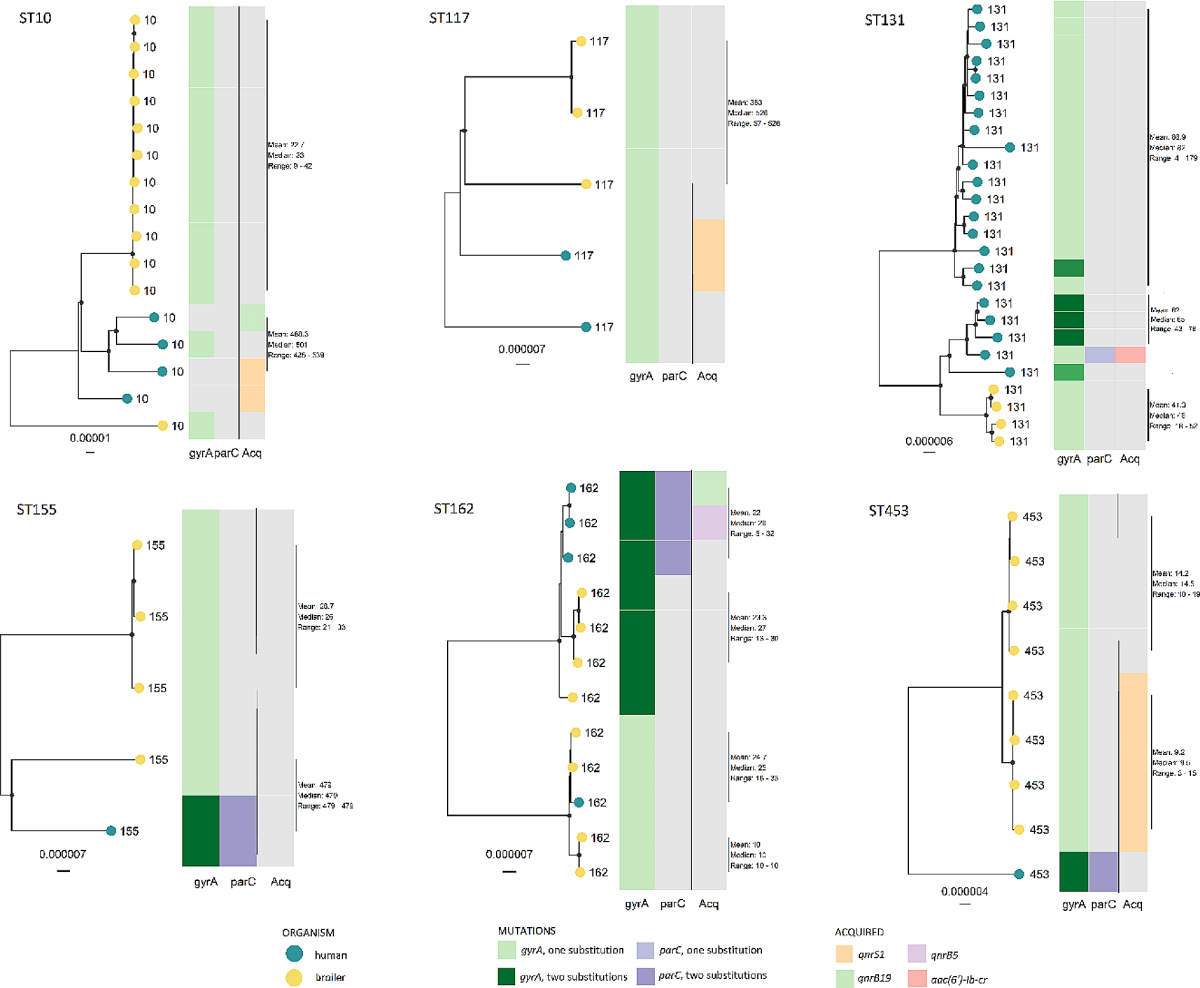
Maximum likelihood core gene midpoint rooted SNP trees for clades ST10, ST117, ST131, ST155, ST162 and ST453. These six clades comprise quinolone-resistant *Escherichia coli* isolates from both humans and broiler. Core genome SNPs were identified with ParSNP, and recombinant sites were removed with Gubbins. ST - sequence type. Genotypes explaining the quinolone resistance mechanism are represented in the heat map

Core genome cluster analyses was performed for the STs that contained either five isolates or more, or isolates from both poultry and humans; they were ST10, ST117, ST131, ST155, ST162 and ST453 (Fig. 3). Within this detailed analysis, human and broiler isolates produced distinctly separate clades with the same resistance mechanisms present for all STs, except for ST162. The average genome coverage, SNP range and SNP distance are described for each of the core genome alignments in Table 3.

**Table 3:** Phylogenetic analysis per sequence type including quinolone-resistant *Escherichia coli* isolates from both broiler and humans

Sequence type (ST)	Number of isolates per species (broiler / human)	Average genome coverage*	SNP range	Median SNP distance	Mean SNP distance
ST10	12 / 4	75.8%	9–1498	512.5	440.0
ST117	3 / 2	85.4%	37–630	526	505.7
ST131	4 / 22	80.8%	4–534	231	265.9
ST155	4 / 1	83.6%	21–588	520	387.9
ST162	8 / 4	89.3%	5–605	538	324.1
ST453	8 / 1	83.6%	3–359	38	99.8

*Average genome coverage is the shared genome found in all isolates included as reported by ParSNP

Within ST162, one of the human QREC isolates clustered with two broiler isolates. This subcluster had an SNP-range of 16–33, a mean SNP distance of 24.7 and a median SNP distance of 25.

## Discussion

In this study, we explored to what extent the QREC population from broilers in Norway could have contributed to QREC in humans. To this end, a dataset comprising QREC isolates from broiler meat and caecum, and from healthy humans and from patients with bacteraemia or UTIs was sequenced and compared. In a large-scale phylogenetic comparison based on gene alignment, it was clear that there was no substantial intermingling between broiler and human isolates. However, some STs did contain isolates that could seem similar, and they were further explored with a phylogenetic tree based on whole genome alignments, applying a threshold of around 20 SNPs, which is a commonly used threshold for source attribution [32]. As is clear from several of the subtrees created, only two contained subgroups of isolates that came close to the criterion; that is ST162 and ST453. When exploring relatedness of isolate sequences there should not only be a low number of SNP differences, but the isolates in question should also be found inside a monophyletic group [32]. This is clearly not the case for ST453. On the other hand, ST162 does have one such group, where one of the human ST162 QREC isolates clustered closely together with two of the broiler isolates (one from caecum and one from meat, with 25 SNP differences to the closest broiler isolate). A limitation that is often left out when comparing sequences, though is how much of the genomes are actually included [33]. For the STs comprising both human and broiler isolates, the average genome coverage ranged from 75.2 to 89.3%. The ST containing a potential monophyletic group with low SNP distance had the highest genome coverage. This means that more than 10% of the genome was not similar enough to be included in the comparison. *E. coli* ST162, which had the highest average genome coverage of 89.3%, included four human and nine broiler QREC isolates. Our finding is supported by a British study where fluoroquinolone-resistant *E. coli* from cattle were compared with those causing bacteriuria in humans living in the same geographical area [34]. They identified possible farm-to-human sharing of ST744 and ST162 but concluded that they had limited impact on community bacteriuria.

However, a clear limitation to our study is that we have no data on level of exposure among humans to broilers or chicken meat. In addition, we did not perform further analyses on these strains collected from 2012 to 2016 regarding PMQR genes, plasmid characterization and more detailed phylogeny, since chromosomal point mutations appeared to be the major mechanism behind quinolone resistance in *E. coli* from both human and broiler.

The results in the present study did not point to broilers as a major source for QREC in humans. Both differences and similarities in STs between humans and broiler were however, detected. There was a higher diversity of STs in human QREC isolates as compared to those from broiler. Also, within some STs, such as ST355, ST453, ST131, and ST393, quinolone resistance mechanisms seemed to be more uniform than for other STs. QREC isolates assigned to ST355 and ST453 all, but one human ST453 isolate, derived from broiler. ST131 and ST393 QREC isolates derived solely from human isolates, except four broiler meat QREC isolates that were assigned in ST131. Similar findings of *E. coli* ST355 were also documented by Röderova et al. [35], who reported ST355 to be one of the major clones prevalent in Czech retail turkeys carrying *qnrS1* and *qnrS1* together with *qnrB19*. In contrast to our study, Ramadan et al. [36], found that *E. coli* isolates from chicken in Egypt were more diverse regarding STs compared to *E. coli* isolates from humans and beef. Our findings are not surprising since the broiler production in Norway is dependent on import of breeding animals originating from a common ancestor [37].

Some of the STs identified in this study, ST10, ST69, ST117 and ST131, belong to successful clones disseminated worldwide and have been detected in antimicrobial resistant lineages of ExPEC from humans and from food/animal sources [22, 24, 26–29, 38]. Human *E. coli* isolates including a sublineage of ST131, namely ST131-*H*30, are known to be multidrug resistant (MDR) [22, 25, 38]. This is somewhat consistent with our study where 15 of the human ST131 QREC isolates were considered MDR, of which two of the four ST131-*H*30 found were MDR. Five ST131 QREC isolates were found in healthy carriers where three were identified as sublineage ST131-*H*30 (data not shown). Manges [24] suggested that *E. coli* ST131 from broiler may be a possible reservoir for human ExPEC, however, in our study this does not seem to be the case as they are phylogenetically distant and the resistance patterns are different. In contrast to our study, a comparable Algerian study demonstrated clonal relationship between ciprofloxacin-resistant *E. coli* ST131 strains in samples from human and broiler [39]. Liu et al. [40] investigated broiler meat samples and clinical cultures for one year and their findings suggested that a ST131-*H*22 sublineage has been established in food of animal origin. This corresponds well to our study where the four broiler isolates belonged to ST131-*H*22 while most of our ST131 human isolates belonged to ST131-*H*41 (*n* = 17 of 22 ST131 from human) (data not shown). Interestingly, the ST131-*H*41 sublineage has been described in *E. coli* isolated from UTIs and related to extended-cephalosporin resistance [41, 42].

When considering the main mechanisms behind quinolone resistance in both broiler and human QREC isolates, the most frequently seen were mutations in the QRDR of the chromosomally located *gyrA* and *parC* genes, though PMQR was also identified. Our results are in concordance with the findings in Börjesson et al. [43] who investigated imported breeding animals to the Swedish broiler production chain and identified that the main mechanism for QREC was due to a single mutation in *gyrA*. Others have reported that chromosomal point mutations are the main mechanisms of QREC in the broiler production chain [28, 44–46]. Studies on other animals such as cats, dogs, pigs and cattle support that chromosomal point mutations in QRDR is the most commonly identified key mechanism [47–50]. However, in some of these studies PMQR genes were also identified, most frequently a *qnr* gene variant [47, 48, 50].

Only a few studies have characterized PMQR genes from *E. coli* from broilers in Europe. In our study, PMQR genes were detected in QREC from 5% of the broiler samples, *qnrS1* (*n* = 4, all ST453) and *qnrB19* (*n* = 1, all ST349), and always together with the point mutation GyrA S83L. Similar findings in broilers have been described in an Italian study of *E. coli* isolated from poultry flocks with colibacillosis [51]. In contrast to our study on broilers, a Czech study on turkeys detected PMQR genes, mainly *qnrB* and *qnrS*, in as much as 58% of the *E. coli* [52]. A recent study from Nigeria detected, similar to our study, no clonal relationship between commensal PMQR carrying *E. coli* isolates from poultry, poultry workers, and poultry farms and market environments [53].

## Conclusion

Based on data from this study, QREC from broiler makes a limited contribution to the incidence of QREC in humans in Norway. There was some relatedness between QREC isolated from humans and broilers, but little was shared across the two groups. Still, some overlap was seen of QREC isolates with the same STs isolated from broiler and humans, but overall, the SNP distance was too high to suggest substantial transfer. Some of the broiler QREC isolates with the same STs as human isolates, belonged to phylogroups that were closely related to human ExPEC, namely B2 and D, but they were not closely related. Only one human QREC isolate, *E. coli* ST162 carrying GyrA S83L substitution, clustered closely to two broiler QREC isolates. Further, the major quinolone resistance mechanisms were chromosomal point mutations in *gyrA* and *parC*. Overall, only a few PMQR genes were detected in the included isolates. These results demonstrate that the human and broiler QREC isolates are not highly related, and that the high occurrence of quinolone resistance is not horizontally transferable. In four of the five broiler QREC isolates carrying PMQR genes, the *qnrS1* gene was detected in *E. coli* ST453. More studies are needed using newer technology like long read sequencing to look at the location of these *qnr*-genes and possible plasmid epidemiology to see if the same plasmids can be found in both human and broiler isolates.

## Materials and methods

### Bacterial isolates

#### Broiler isolates

A selective method for screening for quinolone-resistant *E. coli* (QREC) was implemented on broiler samples in NORM-VET in 2014, resulting in QREC isolates available from 89.5% of caecal flock samples and 70.7% of chicken meat samples. This collection of isolates consisted of 188 caecal and 140 meat isolates [8] and was susceptibility tested under the auspices of the NORM-VET programme. The isolates were classified as QREC with MICs for nalidixic acid and/or ciprofloxacin above ECOFF (EUCAST accessed 04.05.2022, i.e. ciprofloxacin MIC > 0.06 mg/L and/or nalidixic acid MIC > 8 mg/L).

The phylogroup of all 328 isolates was found using the method by Clermont et al. [54] and as described in Mo et al. [55]. Based on phylogroups, MIC values for ciprofloxacin and nalidixic acid, and resistance profile beyond quinolones, the isolates were divided into 86 different groups. Isolates were selected from this list based on their proportional distribution in the dataset, and to have at least one isolate per group. Overall, 47 of the 86 groups had only one isolate, all of which were included. The remaining 53 isolates were randomly chosen from the remaining groups proportionally to the frequency of isolates per group. A total of 100 QREC isolates from chicken meat (*n* = 47) and broiler caecal flock samples (*n* = 53) were included for further analyses in the present study. The broiler isolates metadata is described in Additional file 1.

### Human isolates

A total of 100 human QREC isolates were included. The collection consisted of isolates from UTI and bacteraemia cases (*n* = 77) in 2012–2014 and retrieved through NORM. Further, 23 isolates were included from a study performed at the Norwegian Institute for Public Health (NIPH) from 2014 to 2016 where healthy carriers were screened for the occurrence of QREC [56].

The human isolates were originally susceptibility tested by the disk diffusion method. For full comparability to the broiler isolates, human isolates were retested using the broth microdilution method applied in NORM-VET, and with the Sensititre™ TREK EUVSEC plate (Trek Diagnostic System Ltd., United Kingdom). The EUVSEC panel contains the following 14 antimicrobial agents: ampicillin, azithromycin, cefotaxime, ceftazidime, chloramphenicol, ciprofloxacin, colistin, gentamicin, meropenem, nalidixic acid, sulfamethoxazole, tetracycline, tigecycline, and trimethoprim. The fully susceptible *E. coli* ATCC 25,922 was included as quality control. ECOFFs (EUCAST accessed 04.05.2022) were used to categorize the isolates as susceptible or resistant. The human isolates metadata is described in Additional file 1.

### Whole genome sequencing

Bacteria were inoculated in Luria Bertani (LB) broth (Merck, Darmstadt, Germany) and incubated at 37 °C for 19–21 h in a shaker at 180 rpm. Genomic DNA was extracted through either automatic DNA extraction with QIASymphony DSP DNA Mini Kit or by manual extraction with DNeasy Blood and Tissue kit (both QIAGEN, Hilden, Germany), and according to the producer’s protocols. The DNA concentration and quality were measured using a Qubit™ 4 Fluorometer with Qubit™ dsDNA BR (Broad-Range) Assay kit (Thermo Fisher Scientific) and NanoDrop™ One Spectrophotometer (Thermo Fisher Scientific).

Library preparation and sequencing of the isolates were done at the Norwegian Sequencing Centre [57] using Illumina™ Nextera XT library prep (Illumina, Inc., San Diego, California, USA) on an Illumina™ HiSeq 2500 (Illumina) with Rapid Run spiked with PhiX generating 250 bp paired-end reads with four reads per isolate.

### Assembly and characterization

Analyses were performed using the Bifrost pipeline (10.5281/zenodo.4043861). Specifically, quality control was done using FastQC v0.11.9 [58] (http://www.bioinformatics.babraham.ac.uk/projects/fastqc/), and MultiQC v1.9 [59] to collate the FastQC data. Contamination screening was performed using Mash Screen v2.1 [60]. Genome assembly was performed using Trimmomatic v0.39 [61], followed by SPAdes assembly v3.14.0 [62] with parameters coverage cutoff set to “auto”, “--careful” settings and excluding contigs shorter than 500 nucleotides. The SPAdes assemblies were further run through Pilon v1.23 [63] for finishing, before genome annotation with Prokka v1.14.5 [64]. QUAST v5.0.2 [65] was used for evaluating the assemblies.

Multi-locus sequence typing (MLST) and detection of antimicrobial resistance genes were done using ARIBA [66]. The scheme hosted by Enterobase was used for MLST [23], while the MEGARes [67] and ResFinder (accessed 28 March 2019) [68, 69] databases were used for the detection of ARGs (covering both chromosomal point mutations and acquired, respectively).

### Phylogenetic analysis

An eBURST analysis [70, 71] of the MLST results was performed to do a preliminary investigation of the clonal relationship of the QREC isolates using PHYLOViZ v 2.0 [72]. Further, the genomes were used as input for pangenome analyses using the core gene track in the phylogeny pipeline ALPPACA v0.4.1 [73]. In short, the core genes were identified in the assemblies by running Prokka v1.14.5 [64]. Panaroo v1.2.2 [74] was run in sensitive mode to build a graphical representation of the pangenome that were used to error correct the annotated genomes. SeqKit [75] was used to remove duplicated genomes and SNPs were filtered with *SNP-sites* v2.5.1 [76]. A maximum likelihood (ML) tree was generated using IQtree v1.6.12 [77, 78], and snp-dists v0.6.3 (https://github.com/tseemann/snp-dists) was used to calculate SNP distances.

Genomes belonging to ST groups with more than five isolates from both human and broilers were analysed separately using the core genome track of the ALPPACA v0.4.1 pipeline. In short, the core genome was identified using ParSNP v1.5.3 [79] and duplicated genomes were removed using SeqKit [75]. Gubbins v2.4.1 [80] was used to identify recombinant areas that were further masked with maskrc-svg v0.5 (https://github.com/kwongj/maskrc-svg) before filtering with *SNP-sites* v2.5.1 [76] and generating the ML trees in IQtree v1.6.12 [77, 78].

### Data management

The results from the antimicrobial resistance genes and MLST analysis was collated in R v4.0.5 to produce a summary report. Figures and tables were generated using R v4.0.5 [81], the ggtree package v2.4.2 [82–84] and ggtreeExtra v1.0.4 [85].

### Electronic supplementary material

Below is the link to the electronic supplementary material.

Supplementary Material 1

Supplementary Material 2

Supplementary Material 3

## Data Availability

The dataset supporting the conclusions of this article is available in the European Nucleotide Archive (ENA) repository (https://www.ebi.ac.uk/ena/browser/home), under study accession numbers PRJEB36302 and PRJEB33048, and at Sequence Read Archive (SRA) at National Center for Biotechnology Information (NCBI) (https://www.ncbi.nlm.nih.gov/sra) under BioProduct accession number PRJNA1117742. The dataset supporting the conclusions of this article is included within the article (and its additional file(s)).
